# Supplementary material for: Ammonium Uptake, Mediated by Ammonium Transporters, Mitigates Manganese Toxicity in Duckweed, Spirodela polyrhiza
Source: Plants (Basel). 2023 Jan 3;12(1):208. doi: 10.3390/plants12010208 (PMC9824425; doi:10.3390/plants12010208)
Supplement: Supplementary file 1 [file plants-12-00208-s001.zip › plants-2063286-Supplemental materials.pdf]

Table S1. List of RT-qPCR primers used for evaluating expression of *AMTs aquaporin* genes in *S. polyrhiza* line NB5548.

Gene	Forward primer	Reverse primer	PCR product length, bp
SpAMT1;1	GTTCGTCGCTTATCTCATCT	GGAAAAGGAGGTTGTCGGT	122
SpAMT1;2	TATCTCCTCTTCTCCGCGT	TTCATGGTGTTCTTGGCCC	92
SpAMT1;3	ACCCTCTCTTACTACCTTTT	GAATGCCCACTGGTACAGAA	147
SpAMT2	TCCTGAACACCAACATCTGC	AGTCCCGTCATCATTCCTT	115
SpTIP2;1	TCCATGAACCCTGCTCGCT	GGTAGGCGTAGGTGTAGACAAG	127
SpPIP1;2/SpPIP1;3	TCGGCTTCGCAGTGTTCTT	GGACCTGGACTTGAATGGGA	218
SpPIP2;2	TCGCCACCATCCCTATCA	CACCAATGAAAGGCCCTA	126
SpHiston3	CGCAAGTACCAGAAGAGCAC	CGAAGAGACCCACGAGGTAG	160
SpActin	TGTTTTCCCAAGTATCGTC	TCCCAGTTGGTGACGATT	153

Table S2. List of primer used for cloning *AMT* genes of *S.polyrhiza* ecotype NB5548.

Gene	Forward primer	Reverse primer	PCR product length, bp
SpAMT1;1	AGAGAAGGGAGATGGCGGA	GAGAGAGAAGGTGGGAAGG	1579
SpAMT1;2	GGAGAGATGGGGGACATGG	AAGATATCGATCGGGCCAC	1689
SpAMT1;3	TCTCTCTGCACCTGTCTT	ACAGTTCCCAGTTGTTCC	1453
SpAMT2	GTTGGGGTGAAGAGGATGG	AGGGAGAGAGAGAGACTAGA	1476

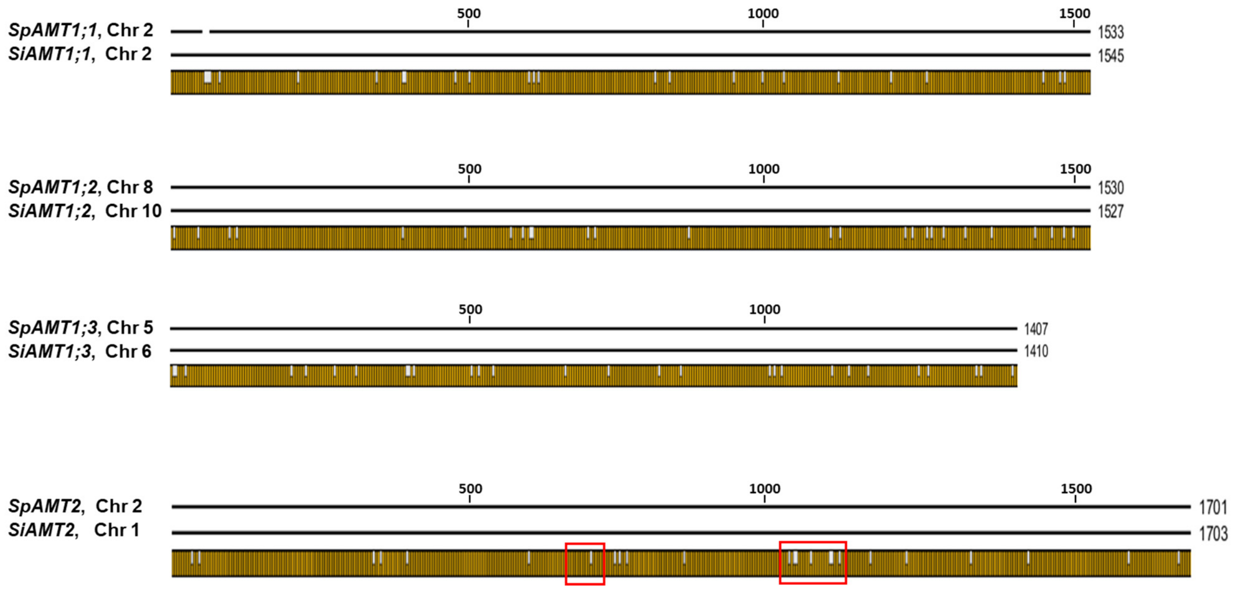

Figure S1. Schematic structure of AMT coding regions in genomes of *Spirodela polyrhiza* (***Sp***) and *Spirodela intermedia* (***Si***). Positions of introns in the *SpAMT2* (643-722 and 1010-1185bp) and *SiAMT2* (643-723 and 1011-1187bp) genes are marked by red boxes.

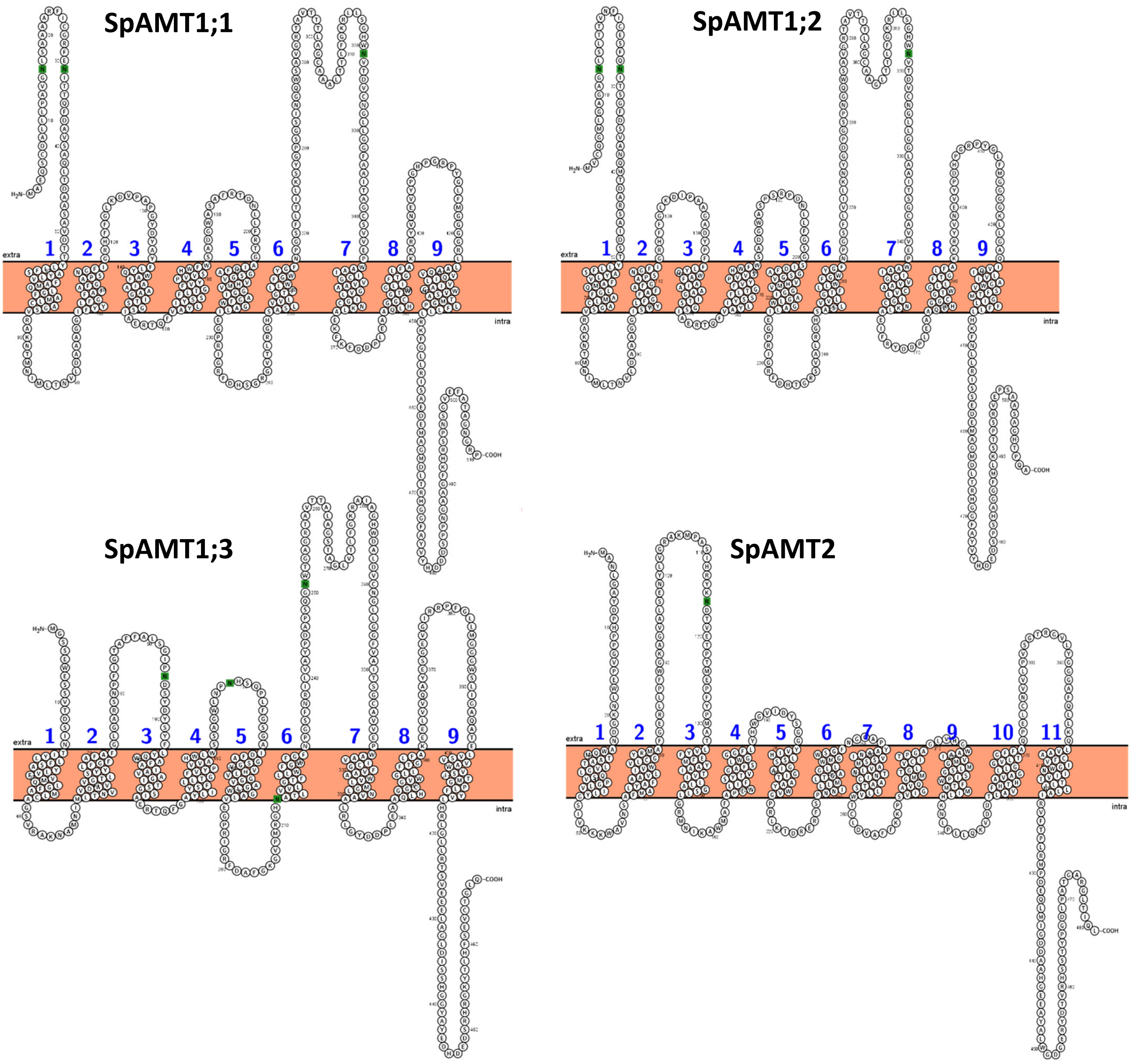

Figure S2. Schematic structure of AMTs of *S. polyrhiza.* Images generated by tool *Protter - visualize proteoforms* [45]

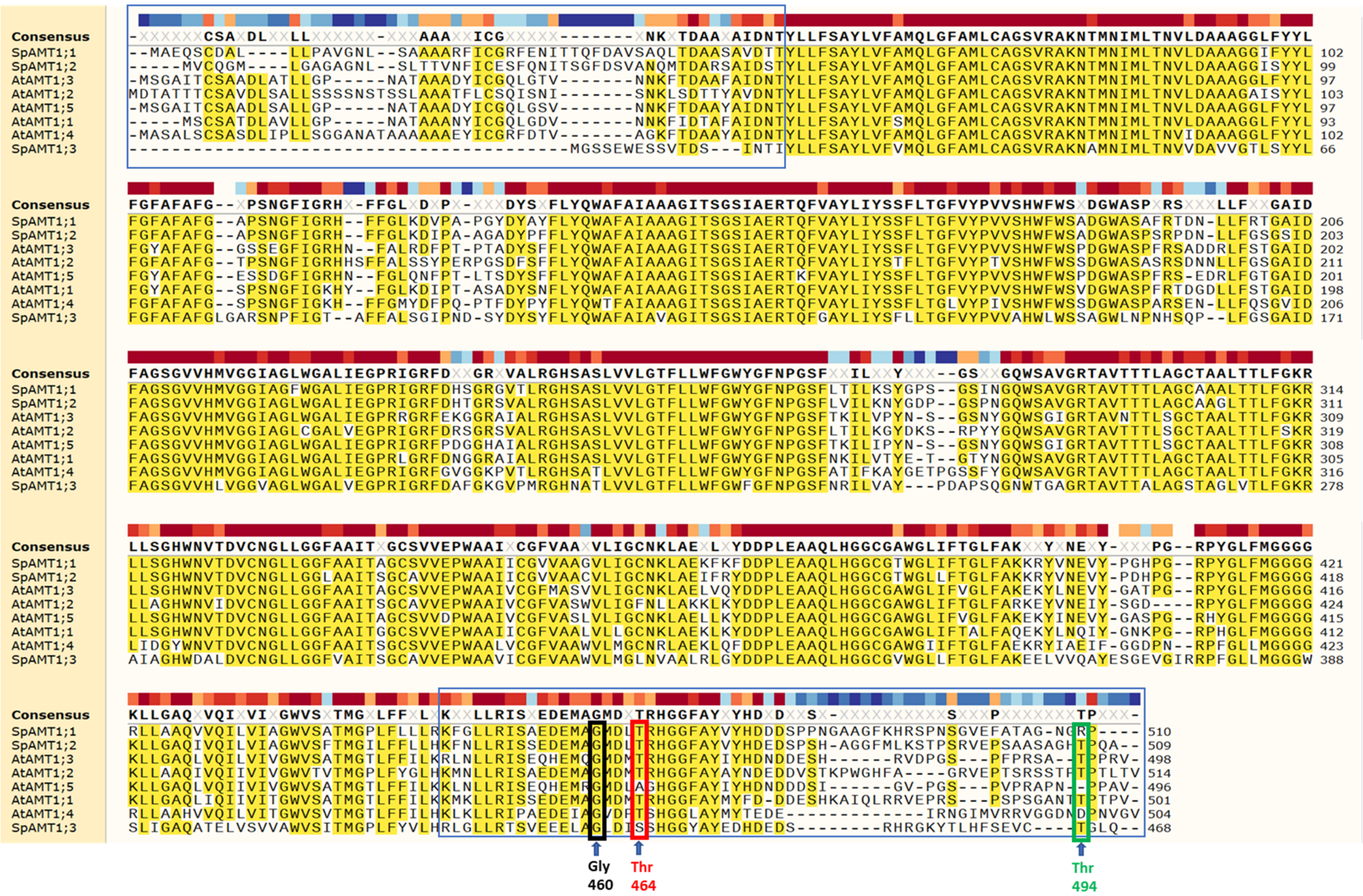

Figure S3. Sequence alignment of AMT1 proteins from *S. polyrhiza* and *A. thaliana*. Gly460, Thr464 and Thr494 reduces labeled according their position in AtAMT1;3. Non-cytoplasmic N-termini domains and the cytoplasmic C-termini domains have been shown in blue boxes.
